# Forces and Straw Cutting Performance of Double Disc Furrow Opener in No-Till Paddy Soil

**DOI:** 10.1371/journal.pone.0119648

**Published:** 2015-03-30

**Authors:** Fiaz Ahmad, Ding Weimin, Ding Qishuo, Mubshar Hussain, Khawar Jabran

**Affiliations:** 1 College of Engineering, Nanjing Agricultural University/Key Laboratory of Intelligent Agricultural Equipment of Jiangsu Province, Nanjing 210031, P.R. China; 2 College of Agriculture, Bahauddin Zakeriya University, Multan, Pakistan; 3 Department of Plant Protection, Adnan Menderes University, Aydin, Turkey; 4 Department of Agronomy, University of Agriculture, Faisalabad, Pakistan; Leibniz-Institute of Vegetable and Ornamental Crops, GERMANY

## Abstract

Conservation tillage is an energy efficient and low cost tillage system to improve soil environment compared with conventional tillage systems. However, the rice residue management becomes an “impossible to achieve” task due to high soil moisture content at harvest time and the thickness of rice straw. Disc type furrow openers are used for both seed drilling as well as straw cutting during no tillage sowing. A study was conducted to evaluate the draft requirement and straw cutting performances of different sized furrow openers in no-till paddy soil conditions. Double disc furrow opener was tested on an in-field traction bench for three working depths, i.e. 30, 60 and 90 mm, and three forwarding speeds, i.e. 0.1, 0.2 and 0.3 m/s. The draft and vertical forces on the disc were recorded with load cells. These sensors were connected to a data acquisition system developed with hardware and software. The results revealed that the size of the furrow opener, operating depth and the forwarding speed had significant effects (P<0.05) on the horizontal and vertical forces, and the straw cutting performance. Mean values of the draft were 648.9, 737.2 and 784.6 N for the opener with diameters of 330, 450 and 600 mm respectively, and the vertical forces for similar openers were 904.7, 1553.9 and 1620.4 N, respectively. Furthermore, the mean straw cutting efficiencies for the double disc opener with diameters of 330, 450 and 600 mm were 39.36, 78.47 and 65.46%, respectively. The opener with 450 mm diameter provided higher straw cutting efficiency as compared to 600 mm diameter disc, while lowest straw cutting efficiency was observed with 330 mm diameter disc. The 450 mm diameter opener provided the highest straw cutting efficiency (88.6%) at 90 mm working depth and expressed optimum performance compared with other furrow openers.

## Introduction

Tillage operations consume a major portion of energy for crop production to modify soil physical, structural and ecological status for a better production of crops [1]. However, conventional tillage systems deteriorate soil structure and thus reduce soil water holding capacity and affect soil biology, having a negative impact on the nutrient supply and storage capacity of soil [2–4]. In contrast, conservation tillage has been reported as an energy efficient and low cost tillage system to obtain higher crop yield [5, 6]. Zero tillage is the extreme form of conservation tillage system which improves input use efficiency, reduces production cost, decreases energy consumption, and finally increases the net benefit of the farmers [6–9]. However, the management of crop residue is a serious constraint in the adoption of conservation tillage due to the mechanical interference of residue in sowing operations, particularly in paddy fields of humid regions [10]. Due to the complexity of soil texture, unique weather conditions, soil genesis and soil structure, paddy soils have a complex nature in term of failure pattern and draft force requirement [11]. Furthermore, puddling operation in paddy fields deteriorates the soil structure and expresses an edaphic conflict in traditional soil management practice for rice and subsequent wheat crop [12, 13]. Thus, for precise direct sowing operations, efficient sowing machinery and effective residue handling is required [14].

Different types of furrow openers are used for conservation tillage systems such as hoe, chisel and disc furrow openers. These openers have their merits and demerits in terms of soil disturbance and force requirements [15]. The disc type furrow openers have lower soil disturbance than the hoe type furrow openers [16, 17]. These are mostly adopted for effective soil failure as well as straw cutting to clean excess straw, which can create problems at the time of sowing. The double disc furrow opener is considered more efficient among the different types of furrow openers [18].

In the past, most studies investigated the performance of furrow openers under low moisture content in soil bins and their effect on soil physical properties [19, 20], seed distribution pattern [21–24] and crop emergence [19, 20, 25–27]. A performance evaluation of different sized double disc furrow openers in term of draft force, vertical force, straw cutting efficiency and cutting phenomena under China’s rice-wheat cropping system in no till paddy field conditions still needs to be elucidated.

The performance of the disc type furrow opener (residue cutting efficiency, draft and vertical forces) is significantly affected by the degree of soil compaction (shear strength and cone index), residue density, coulter type and rotational speed of the furrow opener [28]. Magana et al. [29] investigated the working performance of 425 mm sized notched type and smooth coulters, and concluded that sugarcane residue cutting ability of smooth coulter was lower compared with the notched type coulter. The disc coulters dynamic performance on straw covered soil was reported by Endrerud [30]. The authors argued that the disc coulter penetration depth was influenced by the straw cover. In comparison with notched type and smooth type single disc coulters, toothed type coulter performance was superior for sugarcane residue cutting with low vertical force requirement under soil bin conditions [5] and was in line with the numerical simulation of toothed coulter under control conditions [31]. Hemmat et al. [32] investigated soil mechanical resistance with respect to georeferenced operating depths of a 762 mm diameter disc coulter in various tillage and no-tillage areas. The highest rate of straw cutting is observed with increase in working speed of the coulter [33].

Due to opener geometry and soil-straw conditions, furrow openers generally have problems of low residue cutting capacity. Paddy soil requires a high draft force for tillage [11] and residue is pushed into soil without cutting, causing a hair pinning effect. Therefore, enhancement of tillage efficiency in paddy soil under no till circumstances is still an important challenge. Because of soil complexity, crop residue and tool interactions generally found in Chinese rice-wheat rotations, double disc type furrow opener needs performance evaluation in paddy soil under different working conditions. The present study was conducted with an aim to investigate the draft, reaction forces and straw cutting efficiency of different sized double disc type furrow openers at variable depths and speeds in no-till paddy fields.

## Materials and Methods

### Experimental site

A field experiment was conducted at the Jiangpu Experimental Farm, Nanjing Agricultural University, China (32.34°N and 118.36°E) in a post-paddy soil during December 2013. According to the international soil textural triangle, experimental field soil was clay loam having clay (<0.002 mm) 21.30%, silt (0.2–0.002) 39.84%, sand (>0.2mm) 38.85% and organic matter 3.18%. Soil plastic limit was 26.7% and liquid limit was 47.3%. The soil of the experimental area has a long history of rice-wheat rotation. Soil physical properties are presented in Table 1.

**Table 1:** Soil properties of experimental site and tool parameters.

Soil and tool parameters	Value
Bulk density	1.28 g/cm^3^
Wet density	1.7 g/cm^3^
Soil texture	Clay loam (21.30, 39.84 and 38.85% clay, silt and sand, respectively).
Moisture content of soil	33.34%
Internal friction angle	12.67, 7.70, 8.55° at 0–2, 4–6 and 8–10 cm depth, respectively.
Soil cohesion	42.14, 52.06, 61.68 kPa at0–2, 4–6 and 8–10 depth respectively.
Soil con index	682, 1280, 1000, 1185, 1212 kPa at 0, 2.5, 5, 7.5, 10 cm depth, respectively.
Weight of DD330	10.3 kg
Weight of DD450	19.96 kg
Weight of DD600	25.16 kg

### Description of double disc furrow openers and test bench

**Fig 1:**
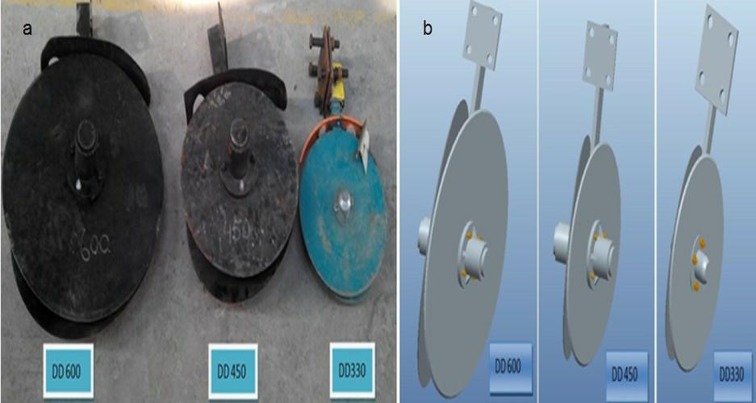
(a) Double disc furrow opener used in study (b) Pro E schematic view of the tested furrow opener.

**Fig 2:**
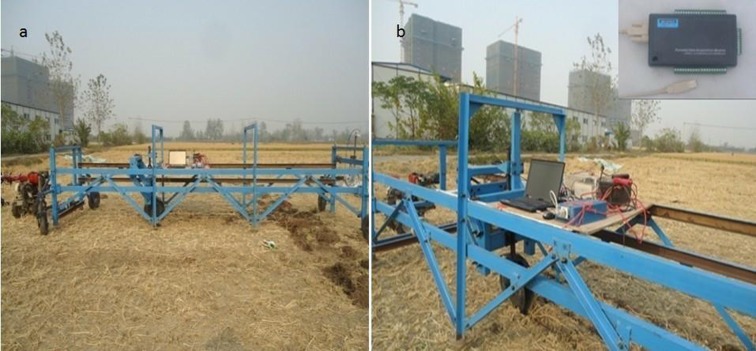
(a) Test bench with power source (b) Data aqusition system with data acquisition card.

**Fig 3:**
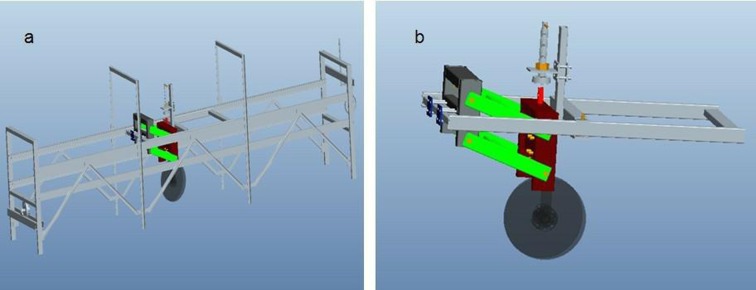
(a) Schematic view of test bench (b) Schematic view of furrow opener adjustment on movable trolley.

Three double disc furrow openers as DD330 (double disc opener with diameter 330 mm), DD450 (double disc opener with diameter 450 mm) and DD600 (double disc opener with diameter 600 mm) were selected for the experiment (Fig. 1 A, B). A test bench designed and developed in the Key Laboratory of Intelligent Agricultural Equipment of Jiangsu Province, Nanjing, China was used to test the performance of the double disc opener in the paddy field (Fig. 2 A, B). Test bench was equipped with a power source, tool moving trolley, tool adjustable frame, and two load transducers to record the horizontal and vertical forces acting on the furrow opener and data acquisition system. The schematic views of the test bench and furrow opener adjustment are presented in Fig. 3 (A, B). The two sensors designated as LSR-2A (2KN, Shanghai, Zhendan Sensor and Instrument Factory, China), were calibrated prior to the experiments and were then installed in the test bench. To measure the draft and vertical forces through sensors, a LabVIEW (National Instruments, Austin, TX, USA) program was developed for data acquisition system. In order to facilitate the data collection, the system used Advantech bus multifunction data acquisition card USB-4711A of sampling frequency up to 150 kS/s.

### Soil physical and mechanical properties

Before the start of experiment, undisturbed soil cores (50 mm diameter and 30 mm long) were collected from the test site. Collected soil cores were brought to the laboratory, weighed, oven-dried at 105°C for 24 h, and then weighed again to measure the soil moisture content (dry basis) and dry bulk density using gravimetric method [34]. Direct shear method was used to determine shear strength, internal friction angle and cohesion [35]. Soil texture was determined using Bouyoucos Hydrometer Method [36–37]. Penetration resistance (cone index) was measured at ten different locations in experimental field using digital penetrometer (TJSD-750, Zhejiang Top Instrument Co. Ltd, China).

### Test procedure

**Fig 4:**
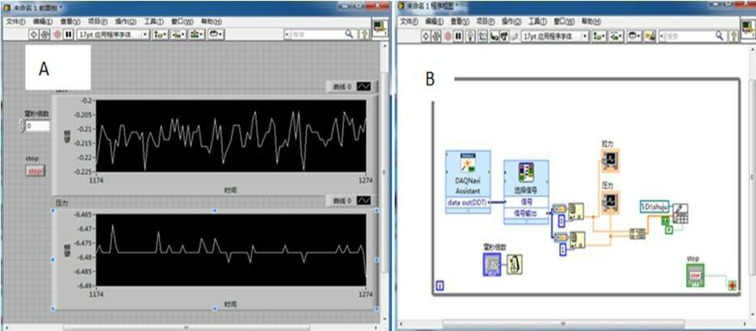
(a) Labview data acquisition front panel (b) Labview data acquisition block.

An experiment with a completely randomized 3^3^ factorial design was carried out at three different sizes of double disc furrow openers (330, 450 and 600 mm), three depths (30, 60 and 90 mm) and at three speeds (01, 0.2 and 0.3 m/s). Each treatment was replicated three times. To examine the straw cutting ability and mechanism, freshly harvested rice straw of 200 g/m^2^ was uniformly spread in front of the furrow opener on the field surface under no-till condition following Kushwaha et al. [28] who applied wheat straw in the soil bin and, Bianchini and Magalhães [5] who used sugarcane residue. Tillage tools were fixed on balance with depth adjustable frame having two pairs of parallel connecting strong bars and frame was then attached to a trolley which could freely move on the test bench (Fig. 3). As the disc tool moved, both draft and vertical forces signal data were recorded through two-channel data acquisition program using the Labview developed model (Fig. 4 A, B). The millisecond voltages collected data was stored in the form of a data table in excel spreadsheets to calculate vertical and horizontal forces on the tool. Simultaneously, straw and soil cutting patterns were recorded using a digital camera (Canon, Canon Inc., China). The recorded videos were then converted to snapshots and analyzed. Furthermore, to determine the straw cutting capacity of the furrow opener after each run of treatment, cut and uncut straw were carefully collected and brought to the laboratory for mass determination and calculations.

### Data analysis

A statistical software package Satistix (version 8.1, Analytical Software, Tallahessee, USA) was used for data analysis. Analysis of variance (ANOVA) was performed to determine the significance of experimental factors (opener diameter, working and depth speed) as well as their interactions. The LSD test was applied to find out a significant difference between the means and pairwise comparison at 5% probability level [38].

## Results and Discussion

Mean values of factors and their levels are presented in Table 2. The data represents the performance of the double disc furrow opener in no- till paddy soil.

**Table 2:** Means of draft, vertical force and straw cutting.

Factors	Levels	Draft (N)	Vertical force (N)	Straw cutting (%)
**Furrow opener**	**DD330**	648.9^a^	904.7^a^	39.4^c^
	**DD450**	737.2^b^	1553.9^b^	78.5^a^
	**DD660**	784.6^c^	1620.4^c^	65.5^b^
**Working depth (mm)**	**30**	403.8^a^	808.8^a^	44.7^c^
	**60**	604.6^b^	1211.1^b^	61.6^b^
	**90**	1162.3^c^	2039.2^c^	76.9^a^
**Working speed (m s^−1^)**	**0.1**	579.6^a^	1107.2^a^	55.2^c^
	**0.2**	739.1^b^	1380.1^b^	60.0^b^
	**0.3**	852.1^c^	1571.7^c^	68.1^a^

Mean values sharing the same superscript letter, for a factor, in a column do not differ significantly at p = 0.05.

### Furrow opener draft force requirement (F_d_) in no-till paddy soil

Tool geometry and implement working conditions had significance effect on soil disturbance and tool force requirements [39]. Soil disturbance characteristics and force requirements are enhanced by the operational depth of the opener [40]. The results of study indicated that the draft force of double disc furrow openers was significantly (*P*<0.05) influenced by the size of furrow opener, working depth and speed in no-till paddy soil (Table 2). The interaction effects of furrow opener × depth and depth × speed on draft force were significant (*P*<0.05). But the interaction effects of furrow opener × speed and furrow opener × depth × speed on draft force were not significant (*P*<0.05). The mean values of draft were 648.9, 737.2 and 784.6 N for DD330, DD450 and DD600, respectively (Table 2), highlighting the increasing draft required to pull larger diameter double disk openers through the soil. The draft force requirement ranged from 365.6 N to 1051.1 N for DD330, 413.3 N to 1175.5 N for DD450 and 432.6 to 1260.2 N for DD600 when the depth of openers was adjusted from 30 to 90 mm (Table 3). At 30 mm depth, the DD330 showed the lower draft force requirement, whereas, DD600 showed the highest draft force requirement at 90 mm operating depth. The mean values of the draft force were 403.8, 604.6 and 1162 N as the working depth varied from 30, 60 and 90 mm, respectively. Kushwaha et al. [28] reported an increase in draft force with an increase in depth for single disc type furrow opener in soil bin conditions. Chaudhuri [15] reviewed the performance of various seed openers and concluded that higher draft force was achieved with increasing working depth. The draft force results showed the similar trend with respect to depth as reported by Bianchini and Magalhães [5]. These researchers reported the horizontal draft of 940 N and 1360 N for single smooth disc type furrow opener to cut the sugarcane residue at 80 and 100 mm working depths, respectively. The larger disc openers had a greater area of contact with the soil and also opened a wider furrow, which led to greater draft. This is in agreement with the existing theory of soil cutting [41]. This theory describes that the tool having higher cutting width need higher draft force. Similarly, based on seven different furrow openers, Darmora and Pandey [42] reported that the draft force is related to tool width. Moreover, Hasimu and Chen [40] observed that the hoe type furrow openers require less draft force and specific draft than the winged hoe type furrow opener. Regarding interactive effects of furrow opener and speed, the lowest draft force (516.86 N) was observed at 0.1 m/s for DD330 while the highest value of draft force (932.6 N) was achieved from DD600 at 0.3 m/s speed (Table 4). The draft force was significantly (p<0.05) influenced by the speed of the disc opener for all levels. Kushwaha et al. [28] reported that the draft force was increased with the increase in rotation speed of coulters. Increase in the draft force with increase in depth and speed is due to high amount of soil resistance and greater soil volume handled with increase in depth, and higher force needed to achieve the soil acceleration with increasing speed [43]. Greater tool penetration was responsible for the increase of soil resistance on the implement.

**Table 3:** Draft and vertical force for furrow opener at various working depth in no-till paddy soil.

Furrow opener	Draft (N)			Vertical force (N)		
	30 mm	60 mm	90 mm	30 mm	60 mm	90 mm
**DD330**	365.6^g^	530^e^	1051.1^c^	574.1^a^	861.1^b^	1279^c^
**DD450**	413.3^gf^	623.3^d^	1175.2^b^	883.1^b^	1336.7^cd^	2381^e^
**DD600**	432.6^f^	660.4^d^	1260.8^a^	969.2^b^	1435.4^d^	2456.7^e^

Means with dissimilar superscripts letters for each type of draft and vertical force are significantly different (P> 0.05).

**Table 4:** Draft and vertical force for furrow opener at various speed depths in no-till paddy soil.

Furrow opener	Draft (N)			Vertical force (N)		
	0.1 m/s	0.2 m/s	0.3 m/s	0.1 m/s	0.2 m/s	0.3 m/s
**DD330**	516.85^f^	659.61^d^	770.3^c^	731.6^a^	963.4^b^	1019.1^b^
**DD450**	598.53^e^	760.15^c^	853.13^b^	1274.2^c^	1550.7^d^	1767.7^e^
**DD600**	623.28^de^	737.5^c^	932.96^a^	1315.7^c^	1626.2^d^	1919.2^f^

Means with dissimilar superscripts letters for each type of draft and vertical force are significantly different (P>0.05)

### Furrow opener vertical force requirement (F_v_) in no- till paddy soil

According to the analysis of variance (ANOVA) test of recorded data, the vertical force was also significantly increased by (p<0.05) diameter of furrow openers, working depth and speed (Table 2). The mean values of the vertical draft force were 904.7, 1553.9 and 1620.4 for DD330, DD450 and DD600, respectively. Vertical force (Fv) increased as a result of an increased diameter of the openers. Similar results had been described for rolling coulters [44], for different diameter of the single disc opener [28], and for various diameters of toothed openers [31]. The interactive effects of furrow opener and depth, furrow opener and speed on vertical force were significant (*P*<0.05) (Tables 3 and 4). The vertical force varied from 574.1 to 1239 N for DD330, 883.1 to 2381 N for DD450 and 969.2 to 2456.7 N for DD 600 under the adjusted depth of tools from 30 mm to 90 mm. The results showed that with increasing the tillage depth, vertical resistance on the tillage tool was increased. The penetration resistance increased in lower layers of soil in paddy field. Puddling in the paddy field changes the soil structure [6, 12, 45]. Bianchini and Magalhães [5] reported the change of the vertical force from 3540 N to 3720 N as the working depth varied from 80 to 100 mm for single disc opener. The mean values of the vertical force were 808.8, 1211.1 and 2039.2 N for the working depth of 30, 60 and 90 mm, respectively. At 30 mm depth, DD330 showed the lower vertical force, whereas, DD600 showed the highest vertical force at 90 mm operating depth. With respect to interactive effect, DD600 expressed highest vertical force 1919.2 N at speed of 0.3 m/s where as DD330 showed lowest vertical force at speed 0.1 m/s (Tables 3 and 4). Kushwaha et al. [28] reported similar type of results and concluded that vertical force increases with the rotational speed of the disc type furrow openers. The vertical load necessary for soil penetration at 70 mm working depth demanded by the toothed disc coulter varied between 1.5 to 2.1 kN in sugarcane residue covered soil [31]. Karayel and Sarauskis [24] concluded that the emergence percentage of maize was highest at downward forces of 1150 and 1400 N due to the best sowing uniformity.

**Fig 5:**
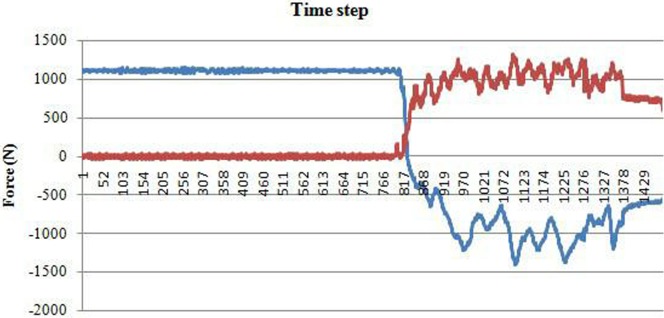
Typical nature of the draft and vertical force on the double disc furrow opener in paddy soil. The blue line represents vertical force and red line corresponds to the draft force.

Furthermore, the typical nature of the draft and vertical force on the double disc furrow opener in paddy soil at plastic phase (moisture content 33.34%) is presented in the Fig. 5. The observed cyclic nature of the draft forces in field conditions is similar to that of in the previous studies at different moisture contents and consistency limits [11, 46] in soil bin conditions.

### Straw cutting efficiency

**Fig 6:**
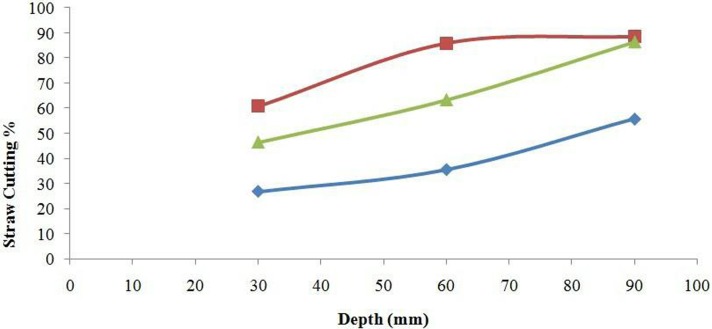
Effect of depth and furrow opener on the straw cutting (%). The values of DD330, DD450 and DD600 are represented by blue diamonds, red squares and green triangles respectively.

**Fig 7:**
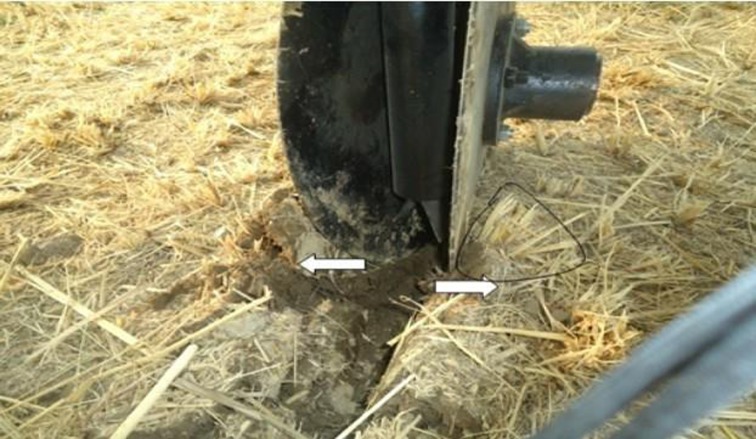
Soil movement and furrow opening mechanism by double disc opener.

**Fig 8:**
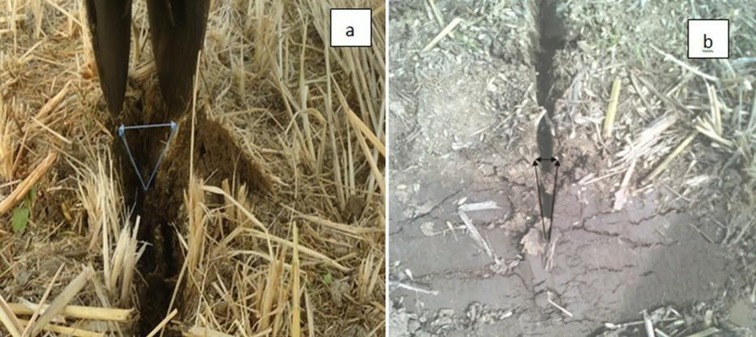
(a) Furrow profile of DD450 (b) Furrow profile of DD330.

The moisture content of straw at the time of experiment was 41.4%. The mechanical properties of the straw (bending strength, shear strength and tensile strength) also affect the cutting performance of furrow openers of all types [28]. According to the LSD test, the straw cutting ability of the furrow openers was significantly affected by disc diameter, working speed and engaging depth. The interaction effects of furrow opener × depth, furrow opener × speed, depth × speed and furrow opener × depth× speed on straw cutting efficiency were significant (*P*<0.05). The DD450 furrow opener showed maximum straw cutting efficiency among all three types of furrow openers with a mean value 78.5% in paddy soil at a soil moisture content of 33.3% for all working depth and speed levels; having the cutting efficiency range from 60.8 to 88.6% while working depth varied from 30 to 90 mm. The DD330 showed straw cutting efficiency with mean values 26.8, 35.5 and 54.7% for working depths of 30, 60 and 90 mm, respectively. The DD600 expressed lower straw cutting efficiency than the DD450 but higher than the DD330 with mean values of 46.6, 63.0 and 86.5% for working depth 30, 60 and 90 mm (Fig. 6). The straw cutting by double disc furrow opener was attributed to the application of tensile force by opener’s disc on the straw and soil (Fig. 7). Furthermore, Fig. 8 (A, B) illustrates the furrow shape developed by the DD450 and DD330 in paddy soil.

By increasing the diameter of the double disc furrow opener from 450 to 600 mm, probably the magnitude of tensile force on straw was reduced and the disc pair of the DD600 furrow opener inserted the more bending and shear forces as compared with the tensile forces on the straw and soil which increased the amount of un-cut straw and increased the hair pinning. Previous studies [5, 28] concluded that the smooth disc type furrow opener cut the deposited residue by a simple shearing rolling acting and the soil act as counter knife. Soil does not provide enough support to hold the straw in position during cutting process, and hence the uncut straw is pushed into soil by the compressive action of the furrow opener. When straw has high moisture content, it is easy to push the straw in to the soil without cutting. The process of hair-pinning was facilitated by high-moisture content of the crop residue which made it easier to fold, allowing the coulter to push the residue into the soil without being cut.

**Fig 9:**
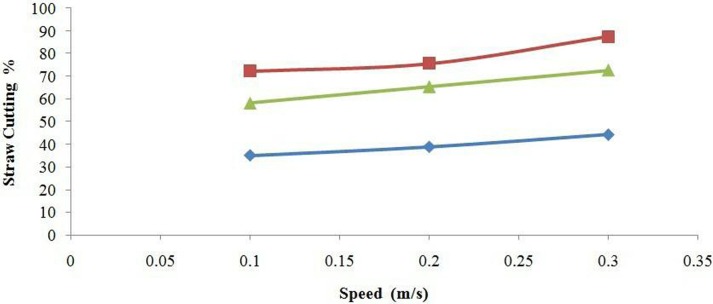
Effect of speed and furrow opener on the straw cutting (%). The values of DD330, DD450 and DD600 are represented by blue diamonds, red squares and green triangles respectively.

**Fig 10:**
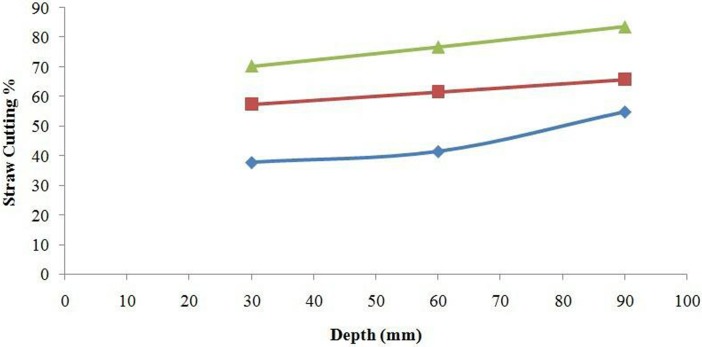
Effect of depth and speed on the straw cutting (%). The blue diamonds, red squares and green triangles correspond to speed of the tool as 0.1m/s, 0.2 m/s and 0.3 m/s respectively.

**Fig 11:**
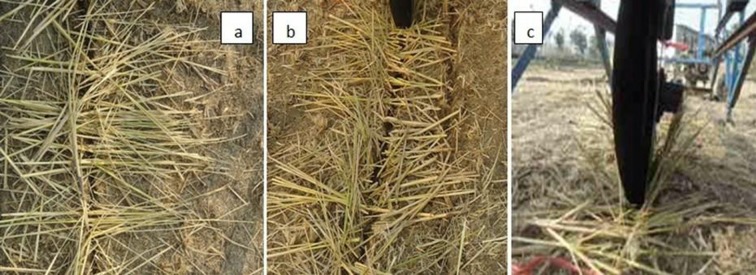
Straw cutting performance of (a) DD330 (b) DD450 (c) DD600.

As the furrow opener speed increased, its straw cutting efficiency also increased. The straw cutting performance of the DD330 (from 35 to 44%), the DD450 (from 72 to 87%) and the DD600 (from 58 to 86%) was measured as the speed changed from 0.1 m/s to 0.3 m/s (Fig. 9). The interaction effect of speed and depth on cutting performance of the furrow openers is presented in Fig. 10. Sarauskis et al. [33] concluded that active disc coulter at highest speeds (speed ratio *λ*>1.27 and *λ* = 1.5) cut larger amount of straw compared with that in the case of inactive disc coulter (*λ* = 1.0). It was found that inactive disc coulters (*λ* = 1.0) cut approximately 30% of the natural moisture (*W* = 10.1%) winter wheat straw while it cut 12% of humid straw (*W* = 22.3%). Magalhães et al. [31] observed that uncut sugarcane residue quantities were statistically similar for the tooth coulter with diameters 508 and 610 mm, whereas the toothed opener with a 711 mm diameter left higher uncut straw than other two openers. Bianchini and Magalhães [5] also reported the significance of different type of furrow opener and working depths for sugarcane residue cutting in sandy clay loam soil under soil bin condition. Fig. 11 (A, B, C) clearly elaborates the difference among the cutting performance of furrow openers i.e. DD330, DD450 and DD600. Superior paddy straw cutting was obtained by furrow opener DD450 compared with the other furrow openers in the experiment i.e. DD330 and DD600. It implies that furrow opener DD450 can be used as an effective straw cutting tool in no-till paddy soil.

## Conclusions

Straw cutting efficiency and soil forces were significantly affected by the opener diameter, working depth and speed in no-till paddy soil conditions. Based on our results, it can be concluded that DD450 showed a superior straw cutting efficiency compared with that of the DD330 and DD600 openers while the opener with diameter 330 mm was noted to have the poorest straw cutting efficiency among the three openers used in this study. On the other hand, the DD330 showed less draft and vertical force than the DD450 and DD600 at all depths and speeds. Therefore, overall it can be concluded that DD450 expressed optimum performance in no-till paddy soil considering straw cutting efficiency, draft force and vertical force. Thus, this study will be useful in making the proper selection of a furrow opener for no-till tillage machinery and its applicability in paddy soil.
